# Improving Emotion Regulation Through Real-Time Neurofeedback Training on the Right Dorsolateral Prefrontal Cortex: Evidence From Behavioral and Brain Network Analyses

**DOI:** 10.3389/fnhum.2021.620342

**Published:** 2021-03-17

**Authors:** Linlin Yu, Quanshan Long, Yancheng Tang, Shouhang Yin, Zijun Chen, Chaozhe Zhu, Antao Chen

**Affiliations:** ^1^Key Laboratory of Cognition and Personality of Ministry of Education, Faculty of Psychology, Southwest University, Chongqing, China; ^2^Faculty of Education, Yunnan Normal University, Kunming, China; ^3^State Key Laboratory of Cognitive Neuroscience and Learning, Beijing Normal University, Beijing, China; ^4^IDG/McGovern Institute for Brain Research, Beijing Normal University, Beijing, China; ^5^Center for Collaboration and Innovation in Brain and Learning Sciences, Beijing Normal University, Beijing, China

**Keywords:** emotion regulation, real-time neurofeedback, right dorsolateral prefrontal cortex, amygdala, resting-state functional connectivity

## Abstract

We investigated if emotion regulation can be improved through self-regulation training on non-emotional brain regions, as well as how to change the brain networks implicated in this process. During the training period, the participants were instructed to up-regulate their right dorsolateral prefrontal cortex (rDLPFC) activity according to real-time functional near-infrared spectroscopy (fNIRS) neurofeedback signals, and there was no emotional element. The results showed that the training significantly increased emotion regulation, resting-state functional connectivity (rsFC) within the emotion regulation network (ERN) and frontoparietal network (FPN), and rsFC between the ERN and amygdala; however, training did not influence the rsFC between the FPN and the amygdala. However, self-regulation training on rDLPFC significantly improved emotion regulation and generally increased the rsFCs within the networks; the rsFC between the ERN and amygdala was also selectively increased. The present study also described a safe approach that may improve emotion regulation through self-regulation training on non-emotional brain regions.

**Highlights**

– Emotion regulation could be improved through self-regulation training on the rDLPFC.
– The training increases the resting-state functional connectivity (rsFC) within the emotion regulation network and frontoparietal network, and the rsFC between the emotion regulation network and the amygdala.
– The training did not influence the rsFC between the frontoparietal network and the amygdala.
– Neural functional connectivity was selectively increased by the training.

## Introduction

Negative emotions, including sadness and anger, are commonly experienced in our daily lives. Failing to regulate negative emotions often adversely affects our social functioning (Murray, 2005; Toni et al., 2008), cognitive abilities (Disner et al., 2011; Miu and Crişan, 2011), and somatopsychic health (Gross, 2015), and can even lead to the development of affective disorders (Beck, 2008; Disner et al., 2011). Hence, the capacity to voluntarily regulate negative emotions is essential to individuals’ normal social and cognitive functioning (Gross, 2015). Given the importance of emotion regulation, many studies have explored methods that improve emotion regulation (Schweizer et al., 2013; Hoorelbeke et al., 2016; Ranney et al., 2017). Among these methods, real-time neurofeedback (rt-nf) training has been used to successfully improve emotion regulation (Linhartova et al., 2019). The rt-nf training is a self-regulation method of brain functioning that displays an individual’s brain activity to themselves through visual, auditory, or other systems and allows them to change their corresponding brain functioning and behaviors (Kamiya, 2011; Sitaram et al., 2017; Papoutsi et al., 2018; Marins et al., 2019). Usually, in emotion regulation rt-nf studies, participants can be instructed to up-regulate or down-regulate their brain activity to increase or decrease negative or positive emotions (Linhartova et al., 2019).

However, participants in previous rt-nf studies were usually required to regulate the activity of target brain regions when their emotions were induced by affective stimuli (Koush et al., 2015; Paret et al., 2016; Nicholson et al., 2018; Herwig et al., 2019) or emotional memory recall (Caria et al., 2007; Young et al., 2018). For example, participants were presented with pictures or scripts with emotional content during NF training periods. Subsequently, they were instructed to regulate the evoked emotions using NF information (Paret et al., 2016). In another approach, participants were instructed to regulate their brain activity by implementing an emotion regulation strategy that recalled their emotional memories without the use of emotion-evoking stimuli (Young et al., 2018). Critically, most previous studies used emotion-related regions, such as the amygdala and anterior insula, as target brain regions (Linhartova et al., 2019). Therefore, the neurofeedback signals reflected both self-regulation and emotion processing. Moreover, rt-nf training with negative stimuli, such as disturbing visuals scenes, may heighten negative emotions in patients with affective disorders (Disner et al., 2011). To avoid these limitations, the present study did not use emotional stimuli or emotional tasks during rt-nf training and selected non-emotional processing brain regions as the target brain regions during training.

Notably, brain regions that are engaged in emotion regulation are also implicated in cognitive control processes (Ochsner and Gross, 2008). Specifically, studies have reported that the dorsolateral prefrontal cortex (DLPFC) is a hub brain region for cognitive control and emotion regulation (Ochsner and Gross, 2005; Ochsner et al., 2012; Buhle et al., 2014; Kohn et al., 2014). That is, there is an interaction between emotion regulation and cognitive control (Ochsner et al., 2002; Ochsner and Gross, 2005); therefore, improving the functionality of prefrontal cognitive control regions may help treat and prevent emotional dysregulation. Moreover, enhancing the function of cognitive control regions (e.g., DLPFC) may directly improve cognitive control for emotion regulation (Feeser et al., 2014). Therefore, we hypothesized that rt-nf training on the DLPFC would improve emotion regulation. However, few studies have investigated whether rt-nf training on the DLPFC improves emotion regulation.

Cognitive reappraisal is a typical strategy that is used for emotion regulation and is usually achieved by changing the initial interpretation of emotional contexts (Ochsner et al., 2002; Gross and John, 2003). On the other hand, the DLPFC is lateralized in processing and regulating emotions: the right DLPFC (rDLPFC) is more involved in the regulation of negative emotion than the left DLPFC (Lévesque et al., 2003; Aboulafia-Brakha et al., 2016; Diefenbach et al., 2016; Notzon et al., 2018). Here, we aimed to examine the facilitation of cognitive reappraisal on negative emotions using real-time- functional near infrared spectroscopy-based neurofeedback (rt-fNIRS-nf) training, which has been shown to improve cognitive control by enhancing the activity of the prefrontal cortex (Barth et al., 2016; Hosseini et al., 2016). Therefore, the current study employed a pretest-training-posttest design whereby negative emotional stimuli were displayed to participants at both pretest and posttest, and the participants were required to regulate their emotion by cognitive reappraisal. Although no emotional stimuli were displayed during the training period and the participants did not need to regulate their emotions, they were asked to regulate the activity of their rDLPFC according to the real-time neurofeedback signal.

Additionally, rest-state functional magnetic resonance imaging (rs-fMRI) data of the participants were recorded at pretest and posttest. The rs-fMRI reflects the spontaneous activity of the brain (Fox and Raichle, 2007), which is independent from emotion regulation and rt-fNIRS-nf training; therefore, it can be used to identify neural changes induced by rt-nf training (Haller et al., 2013; Sitaram et al., 2017; Misaki et al., 2018; Young et al., 2018; Marins et al., 2019). In the present study, we focused on functional connectivity changes of brain networks by comparing changes in resting-state functional connectivity (rsFC) between pretest and posttest. The emotion regulation network (ERN), which is comprised of the DLPFC, ventrolateral prefrontal cortex (VLPFC), inferior frontal gyrus (IFG), superior temporal gyrus (STG), anterior middle cingulate cortex (aMCC), pre-supplementary motor area (SMA), and angular gyrus (AG), subserves emotion regulation (Kohn et al., 2014). Moreover, the DLPFC is a hub brain region in ERN; therefore, we hypothesized that training on this brain region would strengthen the rsFC within the ERN (Bassett and Khambhati, 2017). Further, we hypothesized that training would increase the rsFC between the ERN and amygdala (Ochsner et al., 2012). The DLPFC is also a hub brain region in other brain networks, such as the frontoparietal network (FPN; Cocchi et al., 2013); however, these networks are not involved in emotion regulation. Accordingly, we predicted that training would increase the rsFC within the FPN. We also predicted that training would not modulate the rsFC between the FPN and amygdala.

## Materials and Methods

### Participants

According to Sitaram et al. (2017), a substantial proportion of patients fail to self-regulate specific brain activity during neurofeedback training; therefore we included a session of rt-fNIRS-nf training before the actual experiment to ensure that the selected participants had the ability to self-regulate their rDLPFC activity in the formal experiment. The pre-experiment contained 10 trials (for details, see the “rt-fNIRS-nf Training” section), and the participants who met the pre-experiment test to perform the pretest 1 week after the pre-experiment. Overall, 37 young adult participants were included in the pre-experiment: 24 were included in the neurofeedback (NF) group and 13 were included in the Sham group. Three participants (two participants from the NF group and one participant from the Sham group) were excluded after the pre-experiment because they were not able to successfully self-regulate the activity of the target brain region. Therefore, 34 participants (22 females; mean age = 19.47 years, SD = 1.44 years) passed the pre-experiment and took part in the formal experiment. All of the participants were right-handed and had normal or corrected-to-normal vision. Additionally, none of the participants reported a history of any neurological or psychiatric disorders or had been trained by neurofeedback training before this experiment. Informed written consent was obtained from each participant, and this study was approved by the ethics committee of Southwest University, China. Four participants (three participants from the NF group and one participant from the Sham group) were excluded because they did not complete the entire experiment. Therefore, the final sample consisted of 30 participants: 19 participants in the NF group and 11 participants in the Sham group. *Post hoc* power analysis, calculated by G*Power 3.1 (Faul et al., 2007), suggested that the power was >0.98 when *N* = 30.

### Procedures

#### Emotion Regulation Task

**Figure 1:**
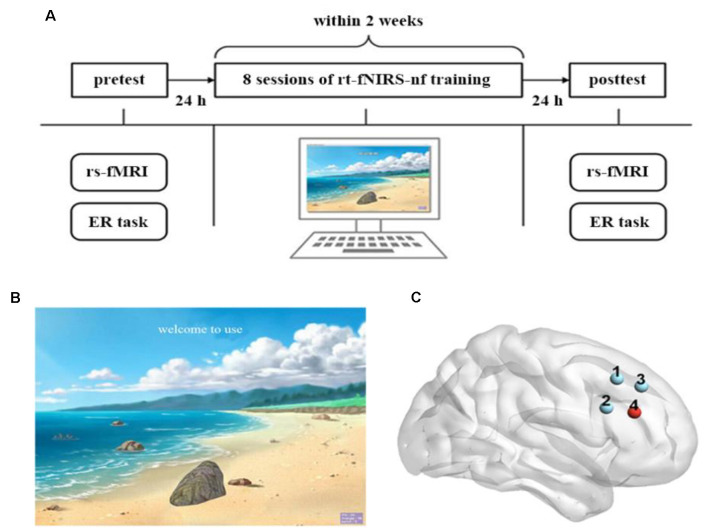
Experimental design. **(A)** The full experimental procedure of each participant. **(B)** The visual feedback interface of the real-time functional near-infrared spectroscopy neurofeedback (rt-fNIRS-nf) training to participants. **(C)** Each dot corresponds to a channel, and channel 4 is the target channel.

The full experiment procedure is displayed in Figure 1A. This experiment consisted of the pretest, rt-fNIRS-nf training (eight sessions), and posttest. All of the participants completed two emotion regulation (ER) tasks at the pretest and posttest (Wager et al., 2008). The resting-state fMRI data of the participants were also recorded before and after the rt-fNIRS-nf training period.

The ER task consisted of 96 aversive images (valence = 2.17, arousal = 5.83) and 48 neutral images (valence = 5.11, arousal = 4.25), which were selected from the Chinese Affective Picture System (CAPS; Bai et al., 2005). These images were randomly divided into two sets under each condition: one for the pretest and the other for the posttest. The two sets were not different in valence (*F*_(1,140)_ = 0.009, *p* = 0.93) or arousal (*F*_(1,140)_ = 0.11, *p* = 0.74; Long et al., 2020). Additionally the participants performed an additional set that consisted of eight aversive images and four neutral images as the practice procedure. Each picture included in the ER task was only used once during the pretest and the posttest.

The task began with one of three cues that represented the following three experimental conditions: (1) Neutral (look neural pictures); (2) Attend (look at negative pictures); and (3) Regulate (cognitively reappraise negative pictures). The participants were instructed to respond to the pictures that were presented following the cue. Specifically, they were asked to rate their emotions in the Neutral and Attend conditions as soon as they saw the corresponding pictures. However, the patients were instructed to rate their emotion after top-down emotion regulation when seeing negative pictures in the Regulate condition.

#### rt-fNIRS-nf Training

During the rt-fNIRS-nf training period, all of the participants received 4 days of neurofeedback training within 2 weeks with an interval of ≥2 days between every two sessions. Each day was comprised of two sessions that contained 10 trials, respectively. Each trial lasted for 1 min: 30 s of rest and 30 s of up-regulation training.

During the rt-fNIRS-nf training period, the participants sat in an electrically shielded and sound attenuated room. Participants were instructed to try their best to regulate the rDLPFC activity according to the visual feedback (the height of the stone on the screen; Figure 1B). They were informed that the goal of this experiment was to lift the stone to the top of the screen, and that they could use several strategies to achieve this goal. For example, they should lift the stone (i.e., up-regulation training) when the green light (represented an up-regulation) appeared on the screen and take a rest (i.e., do not use any strategy) when the red light (represented a rest) appeared. A stone on the screen would be positioned up or down depending on the signal amplitude of the rDLPFC, which was measured by the fNIRS. For instance, the stone would be positioned close to the top of the screen when there was an increase in the signal amplitude of the rDLPFC and close to the bottom of the screen when a decrease occurred. Before the first training period, several strategies without emotional elements, including calculation, spatial imagination, and naming terms of certain categories, were given by the experimenter. Also, participants were encouraged to explore any useful strategies without emotional elements beyond the aforementioned strategies. After reading the instructions shown by the experimenter, a red light was presented to the participants on the screen to indicate the beginning of the experiment. They were also asked to write down all the strategies they had applied after every session, and the responses almost belong to the aforementioned strategies. Moreover, the participants were unfamiliar with the relationship between the height of the stone and the purpose of this experiment until they finished the full experiment. Critically, for the NF group, the height of the stone on the screen corresponded to the actual signal amplitude of the rDLPFC; however, for the Sham group, the feedback signals came from the NF group rather than their own.

#### fMRI Image Acquisition

The resting fMRI data of the participants was recorded with a 3.0 T Siemens Trio scanner before and after the rt-fNIRS-nf training period. Whole-brain functional images were collected using gradient-echo, echo-planar image (EPI) sequences [repetition time (TR) = 2,000 ms, echo time (TE) = 30 ms; flip angle = 90°, FOV read = 220 mm, slice thinness = 3.0 mm, and acquisition matrix = 64 × 64]. The functional images included 32 slices and 242 volumes. Structural imaging consisted of a high-resolution T1-weighted fast-spoiled gradient-echo scan with the following parameters: TR = 1,900 ms, TE = 2.52 ms, flip angle = 9°, FOV read = 250 mm, slice thickness = 1.0 mm, and acquisition matrix = 256 × 256. There were 176 structural image slices obtained.

#### fMRI Data Preprocessing

All of the fMRI data were preprocessed using SPM8 (Welcome Department of Cognitive Neurology, London, UK^1^). The first 10 volumes of each participant were removed to preserve a stable state for subsequent analysis. The remaining data underwent slice-timing correction and were subsequently realigned to the first image in order to correct for head motion. Then, functional data were aligned to T1 data before being normalized to the Montreal Neurological Institute (MNI) template. Additionally, temporal band-pass filtering (0.01–0.08 Hz) was performed to reduce the noise interference. The linear regression was used to remove the artifacts, which included the 24 parameters from head motion correction, the white matter signal, and the cerebrospinal fluid signal. Finally, data were spatially smoothed with a full-width half-maximum Gaussian Kernel of 6 × 6 × 6 mm^3^.

#### fNIRS Data Acquisition and fNIRS Data Online Analysis

We used the FOIRE-3000 NIRS system (Shimadzu, Japan) to measure the relative level of change in oxygenated hemoglobin (O_2_Hb) concentration (Duan et al., 2013; Li et al., 2019). O_2_Hb was used as a feedback signal because it has a better signal-to-noise than deoxygenated hemoglobin (HHb). Moreover, O_2_Hb is more sensitive to changes in itself and increases more robustly during activity than HHb. Therefore, two wavelengths of lights were utilized to quantify the amplitude of O_2_Hb signals: one was 695 ± 20 nm and the other one was 830 ± 20 nm.

In the current study, we used a 2 × 2 probe set that was composed of four channels, two detectors, and two emitters that covered the rDLPFC (Figure 1C). The sampling rate was 25 Hz. We collected and calculated the spatial information of these four channels using the NIRS-SPM toolbox^2^ (based on the MATLAB) to ensure correspondence between the channels with reference to the Broadman’s localization systems. We selected the fourth channel as the target channel because it almost completely corresponds to the rDLPFC (BA46 R). The signal collected in this channel was the source of the feedback signal.

Online preprocessing of the O2Hb NIRS signal was performed by the built-in real-time output solution implemented in the NIRS system. The neurofeedback signals were transmitted to the personal computer through the TCP/IP protocol for computation (a self-programmed MATLAB routine) and presentation. We averaged every 10 data samples into one data point, and the 20 data points that were collected before the first up-regulation were averaged as the baseline. Then, we subtracted every data point from the baseline to obtain the value of the neurofeedback signal. The height of the stone presented on the screen corresponded to the value calculated by dividing the feedback signal by the baseline.

#### fNIRS Data Offline Analysis

After the end of feedback training, the data was analyzed offline to calculate the training effect of the participants during the training phases. First, we used the NIRS-SPM toolbox to pre-process the feedback signal data: band-pass filtering (0.01–0.5 Hz) was conducted to reduce the low-frequency and high-frequency noise. Then, the head motion was corrected using NIRS Analysis Package (Fekete et al., 2011). Subsequently, the data was analyzed by the general linear model (GLM) to compute the beta estimate for each participant of the eight training sessions separately. The GLM approach was employed to model the task-related signal on the individual level, including the up-regulation periods and the rest periods, as an implicit baseline (Li et al., 2019). After computing the beta estimates, we conducted a one-sample *t*-test as the group level analyses to test whether the rDLPFC was activated after rt-fNIRS-nf training sessions of the NF group and the Sham group, separately.

### Statistical Analysis

#### Emotion Regulation Task

We examined the effects of the following three factors in the current study: Group (NF, Sham), Time (pretest, posttest), and Experimental Condition (Attend, Neutral, and Regulate). First, a two-way repeated measure ANOVAs was performed on the pretest results using Group as the between-subjects factor and Experimental Condition as the within-subjects factor to contrast the emotional experience. Then, the experimental conditions were divided into *Emotion Regulation* (Attend, Regulate) and *Emotion Reactivity* (Attend, Neutral; Long et al., 2020). Finally, three-way repeated measure ANOVAs were performed on Emotion Regulation and Emotion Reactivity, respectively, using Group as the between-subjects factor and Time as the within-subjects factor.

#### Resting-State Functional Connectivity Analyses

The region-of-interest (ROI)-wise large-scale brain networks were constructed based on the pre-processed fMRI data. The ROIs in the ERN were selected based on an* a priori* template from a meta-analysis (Kohn et al., 2014) that recruited several brain regions involved in cognitive regulation of emotion tasks across 23 studies and 476 participants. Additionally, we defined the bilateral amygdala ROIs from* a priori* coordinates (Wager et al., 2008). The ROIs of the FPN were selected from the* a priori* template (Power et al., 2011). All of the selected ROIs were placed in a 5-mm sphere, and the center coordinates are summarized in Tables s 1, 2.

**Table 1:** Regions of interest within the emotion regulation network and the bilateral amygdala.

	Region	MNI coordinates		
		*x*	*y*	*z*
Emotion regulation network	Left somatomotor area	−6	14	58
	Left inferior frontal gyrus	−42	22	−6
	Left precentral gyrus	−44	10	46
	Left middle temporal gyrus	−58	−38	−2
	Left angular gyrus	−42	−60	44
	Right somatomotor area	6	14	58
	Right inferior frontal gyrus	50	30	8
	Right precentral gyrus	48	8	48
	Right middle temporal gyrus	38	22	44
	Right angular gyrus	60	54	40
Amygdala	Left	−24	0	23
	Right	21	0	22

**Table 2:** Regions of interest (ROIs) within the frontoparietal network.

	Region (Left)	MNI coordinates			Region (Right)	MNI coordinates		
		*x*	*y*	*z*		*x*	*y*	*z*
Frontoparietal network	Frontal pole	−42	38	21	Frontal pole	24	45	−15
	Frontal pole	−34	55	4	Frontal pole	34	54	−13
	Frontal pole	−42	45	−2	Frontal pole	38	43	15
	Superior frontal gyrus	−23	11	64	Frontal pole	43	49	−2
	Middle frontal gyrus	−41	6	33	Middle frontal gyrus	32	14	56
	Middle frontal gyrus	−42	25	30	Middle frontal gyrus	40	18	40
	Inferior frontal gyrus	−47	11	23	Middle frontal gyrus	48	25	27
	Precentral gyrus	−44	2	46	Precentral gyrus	47	10	33
	Superior parietal lobule	−28	−58	48	Superior parietal lobule	33	−53	44
	Supramarginal gyrus	−53	−49	43	Supramarginal gyrus	49	−42	45
	Angular gyrus	−42	−55	45	Angular gyrus	44	−53	47
	Paracingulate gyrus	−3	26	44	Inferior temporal gyrus	58	−53	−14
					Lateral occipital cortex	37	−65	40

*Note: For each region of interest within the emotion regulation network, frontoparietal network, and the bilateral amygdala, a 5-mm sphere was separately placed around the center coordinate within the left- and right-hemispheres based on the priori templates. MNI, Montreal Neurological Institute standardized space*.

**Figure 2:**
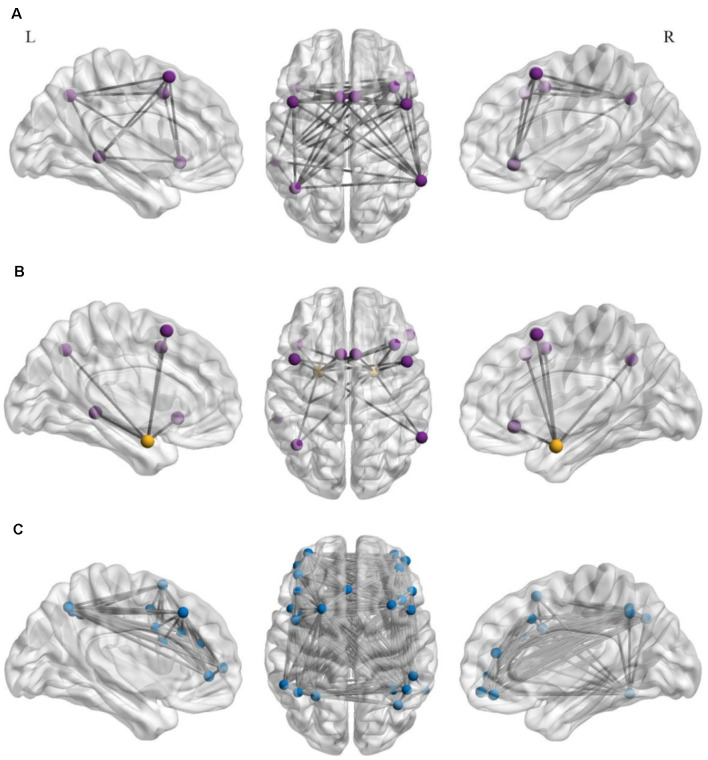
Axial (center) and left (L)- and right (R)-hemisphere sagittal views of the regions of interest (ROIs) for the resting-state functional connectivity in the emotion regulation network (ERN; **A**) resting-state functional connectivity between the ERN and the bilateral amygdala **(B)** and in the frontoparietal brain network (FPN; **C**). Each region of interest was placed in a 5-mm sphere around the center coordinates of peak activation for each discrete cluster within the left- and right- hemispheres. The purple spheres represent the ROIs within the ERN, orange spheres represent the bilateral amygdala **(A,B)**, and blue spheres represent the ROIs within the FPN **(C)**.

Raw time-series data for all voxel contained in each ROI were calculated as the average ROI seed data. Pearson’s correlation coefficients were computed for each ROI with other ROIs, and each correlation coefficient represented connectivity. Then, we converted the *r*-values to *z*-scores using the Fisher’s *z-transformation*. For the ERN, the *z*-scores of all connectivity within the network were averaged to calculate a final value that represented the functional connectivity (Brody et al., 2019; Nusslock et al., 2019). Finally, we computed the *z*-scores between each ROI in the ERN and the bilateral amygdala, and the two averaged *z*-scores reflected the connectivity between the ERN and the left- and right-amygdala, separately. The calculation method used for the FPN was the same as that used for the ERN. Figure 2 presents the axial and sagittal views of the ROIs for each rsFC within the ERN and the FPN and between the ERN and the bilateral amygdala.

## Results

### Emotion Regulation Task

**Figure 3:**
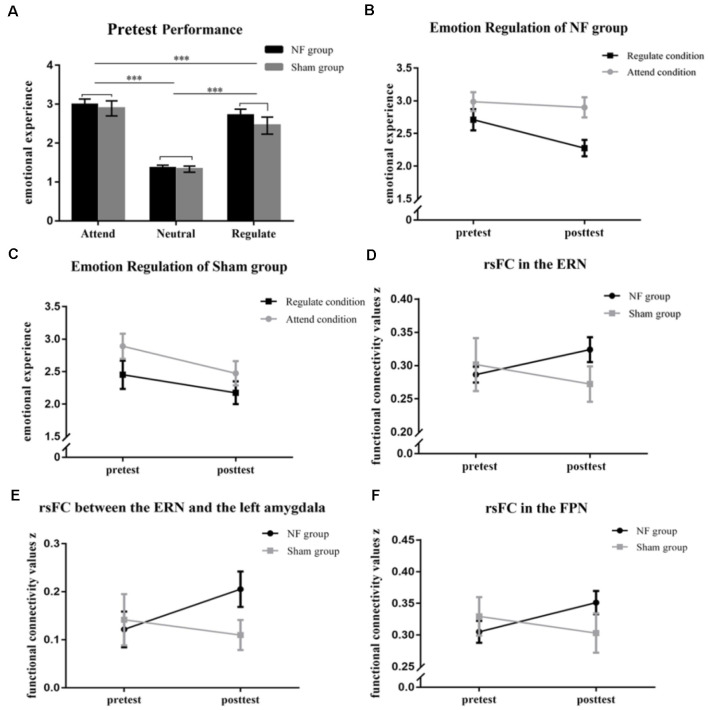
**(A)** The graph shows the emotional rating of the NF and Sham groups at the pretest of the emotion regulation (ER) task. The figures represent the changes in emotional experience in the Regulate and Attend conditions **(B,C)**. The rsFC was significantly enhanced within the ERN **(D)** and between the ERN and the left amygdala **(E)** in the NF group, but not the sham group, across rt-fNIRS-nf training. **(F)** The picture represents the changes in rsFC within the FPN in the NF and Sham groups across rt-fNIRS-nf training. ***p < 0.001.

Pretest performances are shown in Figure 3A. According to our results, participants in the NF and Sham groups had similar emotional experiences. Specifically the main effect of Group (*F*_(1,28)_ = 0.56, *p* = 0.46) and interaction effect of Group × Experimental Condition on pretest performance (*F*_(2,56)_ = 0.52, *p* = 0.60) were not significant. However, there was a significant main effect of Experimental Condition on pretest performance (*F*_(2,56)_ = 103.03, *p* < 0.001 *η*^2^ = 0.79), and the participants experienced more negative emotions during the Attend condition than they experienced during the Neural condition (*p* < 0.001) and Regulate condition (*p* < 0.001).

Results from the three-way repeated measures ANOVA revealed that there was a significant interaction of Group (NF, Sham) × Time (pretest, posttest) × *Emotion Regulation* (Attend, Regulate; *F*_(1,28)_ = 7.25, *p* = 0.012, *η*^2^ = 0.21). The main effects of Time (*F*_(1,28)_ = 19.43, *p* < 0.001, *η*^2^ = 0.41) and *Emotion Regulation* (*F*_(1,28)_ = 30.29, *p* < 0.001, *η*^2^ = 0.52) were extremely significant. Subsequently, we deconstructed the three-way interaction into each group separately. We found that the main effects of Time and Emotion Regulation were significant both in the NF group (Time: *F*_(1,18)_ = 8.81, *p* = 0.008, *η*^2^ = 0.33; *Emotion Regulation*: *F*_(1,18)_ = 20.63, *p* < 0.000, *η*^2^ = 0.53) and the Sham group (Time: *F*_(1,10)_ = 12.43, *p* = 0.005, *η*^2^ = 0.55; *Emotion Regulation*: *F*_(1,10)_ = 15.50, *p* = 0.003, *η*^2^ = 0.61). A significant two-way interaction in the NF group showed that feelings of negativity in the NF group were lower within the Regulate condition than the Attend condition (*F*_(1,18)_ = 13.43, *p* = 0.002, *η*^2^ = 0.43; Figure 3B); however, there were no changes in the Sham group (*F*_(1,10)_ = 0.86, *p* = 0.367; Figure 3C; the rest results of the deconstruction can be seen in the Supplementary Materials and Supplementary Figure 1).

Next, we included Group, Time, and *Emotion Reactivity* (Attend, Neutral) in a three-way repeated measures ANOVA, and the results revealed that the three-way interaction was not significant (*F*_(1,28)_ = 2.25, *p* = 0.145); therefore, changes in emotion reactivity in the NF group and the Sham group were not significant.

### Resting-State Functional Connectivity

Our results indicated that there was significant Group × Time interaction on the rsFC within the ERN (*F*_(1,28)_ = 5.01, *p* = 0.033, *η*^2^ = 0.15; Figure 3D) and between the ERN and the left-amygdala (*F*_(1,28)_ = 4.48, *p* = 0.043, *η*^2^ = 0.14; Figure 3E). However, there was not a significant interaction between the ERN and the right-amygdala (*F*_(1,28)_ = 0.42, *p* = 0.52). We found that the changes in functional connectivity within the ERN (*p* = 0.047) and between the ERN and the left amygdala (*p* = 0.017) induced by the rt-fNIRS-nf training in the NF group were significantly larger than those in the Sham group (within the ERN: *p* = 0.23; between the ERN and the left-amygdala: *p* = 0.47). Furthermore, there was significant Group × Time interaction on the rsFC within the FPN (*F*_(1,28)_ = 5.50, *p* = 0.026, *η*^2^ = 0.16; Figure 3F). The rsFC within the FPN was increased after the rt-fNIRS-nf training in the NF group (*p* = 0.020) while the rsFC within the FPN was not increased after training in the Sham group (*p* = 0.29). However, the rsFC between the FPN and the amygdala did not change across training in the NF group or Sham group (*p* > 0.05).

### Off-Line Analysis of fNIRS Data

All trials of each participant met the head motion correction test. After obtaining the beta estimates by using the GLM for each participant of each session, a one-sample *t*-test was conducted as the group level analyses to test whether the rDLPFC was activated. The results showed that the NF group significantly activated their rDLPFC during each of the rt-fNIRS-nf training sessions; however, the Sham group did not activate the target brain region. The specific statistical values are summarized in Table 3. Besides, in order to evaluate the data quality, the time course of eight fNIRS-based neurofeedback sessions in all the participants of the two group were presented separately in the Supplementary Materials and Supplementary Figure 2.

**Table 3:** Two sided one-sample *t*-test of beta estimates in each session.

Session	NF group		Sham group	
	*t*	*p*	*t*	*p*
1	5.18	0.000***	1.49	0.16
2	4.93	0.000***	0.82	0.43
3	4.02	0.005**	0.61	0.55
4	4.67	0.000***	1.80	0.10
5	4.32	0.002**	0.97	0.35
6	5.59	0.000***	0.15	0.87
7	4.62	0.000***	−0.35	0.97
8	5.08	0.000***	0.98	0.35

Asterisks (*) indicates significant effects after correction for multiple comparison (FDR corrected).

Next, a one-way repeated measures ANOVA was conducted to compare differences in activation between the eight training sessions. Results from the one-way repeated measures ANOVA revealed that the activity of the target brain region did not gradually increase in the NF group during the training sessions (*p* > 0.05).

## Discussion

Using a pretest-intervention-posttest design, the present study combined a cognitive reappraisal task, rt-fNIRS-nf training, and rest-state fMRI measures to investigate the transfer from self-regulation training on the rDLPFC *via* the rt-fNIRS-nf signal to emotion regulation and its neural mechanisms. Behaviorally, emotion regulation did not differ between the NF and Sham groups at pretest; but the regulate effect was significantly higher in the NF group than in the Sham group at posttest. The significant interaction between Group (NF, Sham) and Time (pretest, posttest) verified that the training significantly improved emotion regulation. Correspondingly, the training significantly increased the rsFC within the ERN (and FPN). Further, training also significantly enhanced the rsFC between ERN and amygdala; however, training did not affect the rsFC between the FPN and amygdala.

Previous studies have verified the feasibility of up-regulating the DLPFC activity in neurofeedback training. Sherwood et al. (2016) found that self-regulation enhanced left DLPFC activity and resulted in the improvement of working memory performance in healthy participants. Recently, Kohl et al. (2019) reported that the enhanced activation of the left DLPFC by rt-fMRI-nf training improved self-control in obesity. The authors suggested that the behavioral improvements resulted from the enhancements in cognitive control that was derived from the self-regulation on DLPFC activity (Sherwood et al., 2016; Kohl et al., 2019). Accordingly, the current rt-fNIRS-nf training on the rDLPFC may enhance cognitive control first and subsequently lead to improvement in emotion regulation. Indeed, higher DLPFC activity during cognitive control has been associated with increased utilization of cognitive reappraisal and lower negative emotions and clinical diagnoses (Scult et al., 2017). Moreover, modulating cortical activity in the DLPFC leads to improved cognitive control and emotion regulation in patients with major depression (Salehinejad et al., 2017). Therefore, improvements in emotion regulation may be driven by enhanced cognitive control by self-regulating on the rDLPFC activity through the rt-fNIRS-nf signal, which also indicates the causal engagement of the DLPFC (cognitive control) in emotion regulation.

Further, the functional connectivity analysis showed that the whole rsFC within the ERN was enhanced by the self-regulation training on the rDLPFC. Undoubtedly, the training on the rDLPFC can enhance its own function. Meanwhile, the rDLPFC is a crucial node of the ERN; therefore, increased rsFC within the ERN may be due to enhanced rDLPFC function. Studies have reported that the rDLPFC receives the need to regulate emotion from the VLPFC. Then, it sends a feedforward signal to other ERN nodes (e.g., angular gyrus, pre-SMA, STG) to generate a new emotional state (Kohn et al., 2014). Thus, the rDLPFC is a hub node of the ERN and may coordinate other functions that are necessary in emotion regulation, such as emotion appraisal (VLPFC), action inhibition (aMCC), language (STG), imagination, and social cognition (AG). Importantly, there are anatomical connections between the rDLPFC and other nodes of ERN (Ongur and Price, 2000; Ray and Zald, 2012). Accordingly, training may drive the ERN to reach a higher functional connectivity level *via* the rDLPFC, which contributes to behavioral improvements in emotion regulation. Interestingly, the current results showed that training also increased the rsFC within the FPN, and this finding verified that training on a hub brain region of a brain network can enhance the connectivity efficiency of that network because the rDLPFC is also believed to be a hub node of the FPN (Cocchi et al., 2013).

Although the amygdala is a crucial brain region responsible for emotional processing (Anderson et al., 2003; Cunningham and Brosch, 2012), there is no node in the ERN that directly processes emotion; therefore, the interaction between the limbic circuitry, including the amygdala and ERN, is necessary for the implementation of emotion regulation (Kohn et al., 2014; Paret et al., 2016; Young et al., 2018). Our results showed that rt-fNIRS-nf training significantly increased the rsFC between the ERN and amygdala. Although training significantly increased the rsFC within the FPN, the rsFC between the FPN and amygdala was not modulated by training. Thus, training on the rDLPFC may enhance the rsFCs within brain networks where it plays a role as a hub; however, the enhanced networks facilitate their specific functions using different mechanisms. The increased rsFC between the ERN and amygdala should subserve the improvement in emotion regulation, which may be due to the anatomical connection between the amygdala and some nodes of the ERN (e.g., VLPFC, anterior insula; Ray and Zald, 2012). The current study also revealed how the rDLPFC influences the amygdala even though these two brain regions are not anatomically connected (Hartley and Phelps, 2010; Kohn et al., 2014). Thus, the increased rsFC within the ERN and between the ERN and amygdala convergently contribute to the improvement in cognitive reappraisal by facilitating top-down cognitive control on emotion processing.

Previous rt-nf training studies on emotion regulation usually used the emotion processing regions as target regions and asked participants to practice cognitive reappraisal during the training (Johnston et al., 2010; Koush et al., 2015; Paret et al., 2016; Nicholson et al., 2018; Qiu et al., 2018; Young et al., 2018; Herwig et al., 2019; Linhartova et al., 2019); however, our study used the rDLPFC, which is a hub of cognitive control, as a target region. Moreover, no emotional stimuli were displayed during the training sessions, and the task was not related to emotion processing or regulation. Therefore, the regulation of the target brain region activity during the training sessions should have not evoked emotional responses. Accordingly, the current improvement in emotion regulation may reflect a transfer from the training on cognitive control to emotion processing. Additionally, patients who are diagnosed with affective disorders can also undergo the rt-fNIRS-nf training on the DLPFC (cognitive control) for a relatively long duration. Conversely, the protocols that use emotional brain regions as targets should not be applied to patients with affective disorders because this intervention may worsen their affective disorders (Disner et al., 2011). Thus, the approach established in the current study may improve emotion regulation across a wide range of individuals.

However, the current findings highlight the benefits of rt-fNIRS-nf training on the DLPFC, but there are a few limitations that need to be considered. First, although the final sample in the current study met the *post hoc* power test and the power was >0.98, the proportion of NF and Sham group seems not reasonable enough. Then, after a significant one-sample *t*-test for the offline fNIRS data analysis, we conducted a one-way repeated measures ANOVA to investigate whether there was a learning effect across the eight training sessions of the NF group. However, the result was not significant. This might result from the ceiling effect as our participants mainly involve young college students with high education level and potential above-average frontal control due to their major (Vakhtin et al., 2014). Moreover, the significant activation in DLPFC indicated that the participants in the experimental group did pay attention to the neurofeedback signal and tried to regulate their brain activity. Thus, the participants improved conflict adaptation owing to the benefits of neurofeedback training. But, learning the upper limit of the ability to control brain activity to regulate emotions is a scientifically-interesting question and future work can examine the optimal boundaries of targeted brain region activity in the rt-nf training to best improve emotion regulation ability.

Overall, the present study demonstrated that the rt-fNIRS-nf training protocol, which corresponds to the increased rsFC within the ERN and the increased rsFC between the ERN and the left amygdala, can be used to improve emotion regulation by up-regulating the activity of the rDLPFC. Additionally, these results suggest that the enhanced interaction between cognitive control and emotion processing subserves this improvement. Further, training increased the rsFC within the FPN but did not affect the rsFC between the FPN and left amygdala, which verified that the ERN is exclusively associated with emotion regulation. This study highlighted the causal engagement of the rDLPFC in emotion regulation and showed that self-regulation training on the rDLPFC (cognitive control) *via* the rt-fNIRS-nf signal, which is a neutral and safe intervention, can improve emotion regulation, and this has important clinical implications.

## Data Availability Statement

The raw data supporting the conclusions of this article will be made available by the authors, without undue reservation.

## Ethics Statement

The studies involving human participants were reviewed and approved by the ethics committee of Southwest University, China. The patients/participants provided their written informed consent to participate in this study.

## Author Contributions

LY: conceptualization, software, formal analysis, and writing—original draft. QL: conceptualization, methodology, and visualization. YT: conceptualization, investigation, and data curation. SY: validation and software. ZC: investigation and data curation. CZ: software and methodology. AC: conceptualization, methodology, and writing—review editing. All authors contributed to the article and approved the submitted version.

## Conflict of Interest

The authors declare that the research was conducted in the absence of any commercial or financial relationships that could be construed as a potential conflict of interest.

## Footnotes

^1^https://www.fil.ion.ucl.ac.uk/spm

^2^https://www.nitrc.org/projects/nirs_spm/
