# Supplementary material for: Improving Emotion Regulation Through Real-Time Neurofeedback Training on the Right Dorsolateral Prefrontal Cortex: Evidence From Behavioral and Brain Network Analyses
Source: Front Hum Neurosci. 2021 Mar 17;15:620342. doi: 10.3389/fnhum.2021.620342 (PMC8010650; doi:10.3389/fnhum.2021.620342)
Supplement: Supplementary file 1 [file Data_Sheet_1.docx]

**Supplementary Material**

1.

We deconstructed the three-way interaction (Group (NF, Sham) × Time (pretest, posttest) × Emotion Regulation (Attend, Regulate)) into pretest and posttest separately. For the pretest, the results were equal to the Figure 3A; for the posttest (Figure S1 A), participants in the NF and Sham groups had similar emotional experiences.Specifically the main effect of Group (*F*_(1,28)_ = 1.55, *p* = .22) was not significant.However, the interaction effect of Group × Experimental Condition on pretest performance was marginal significant(*F*_(1,28)_ = 3.25, *p* = .08). Moreover, there was a significant main effect of Experimental Condition on posttest performance (*F*_(1, 28)_ = 26.35, *p*< .001*η^2^*= 0.49), and the participants experienced more negative emotions during the Attend condition than they experienced during the Regulate condition (*p*< .001).

We deconstructed the three-way interaction into Attend condition (Figure S1 B) and Regulate condition(Figure S1 C) separately.For both Attend and Regulate condition, there were significant main effects of Time (Attend: *F*_(1, 28)_ = 10.95, *p*= .003*η^2^*= 0.28; Regulate: *F*_(1, 28)_ = 16.23, *p*< .001*η^2^*= 0.37) while the main effects of Group and the two-way interactions did not (*p*> .05).


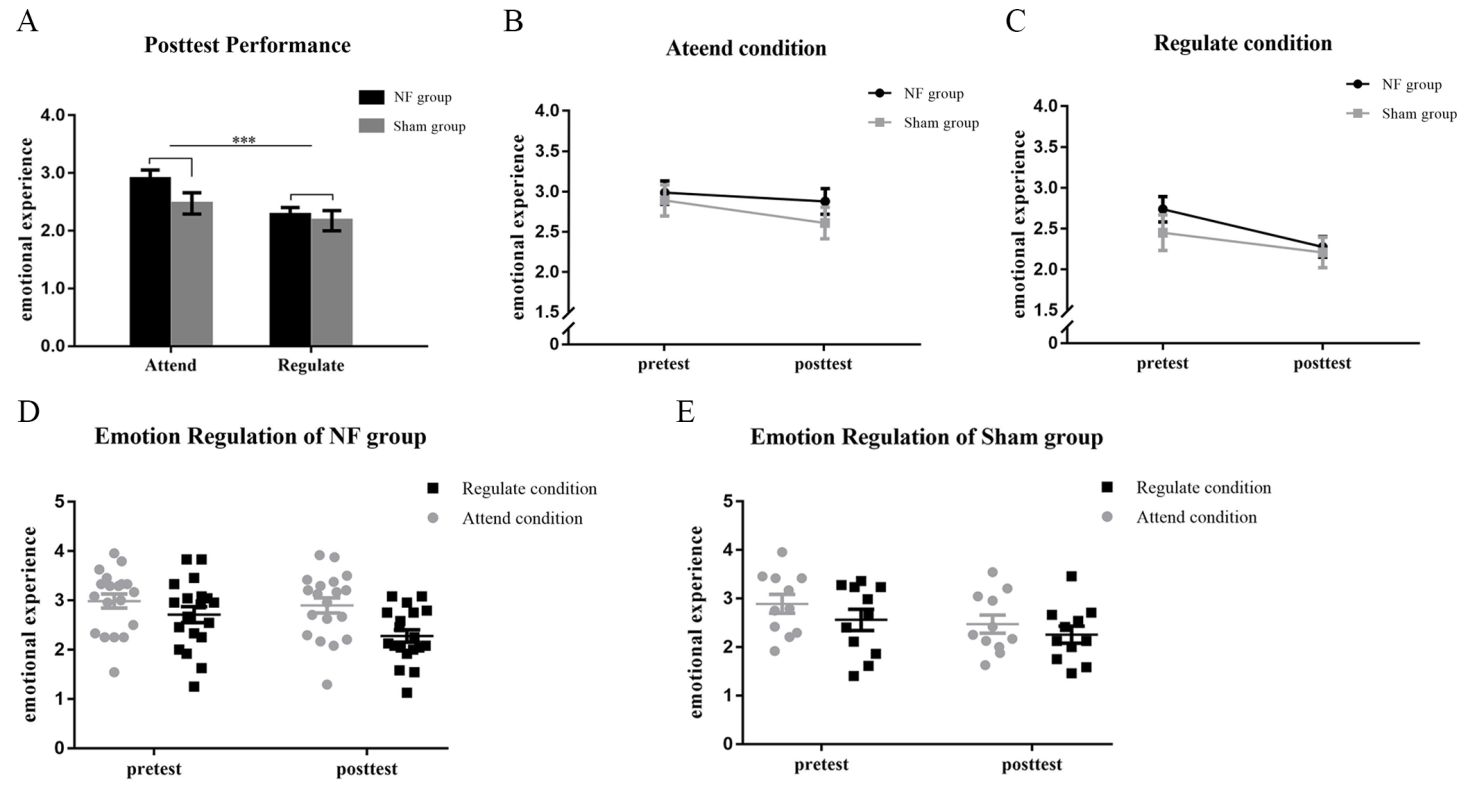


**Figure S1**.(A)The graph shows the emotional rating of the NF and Sham groups at the posttest of the Emotion Regulation condition.The figures represent the changes ofemotional experience ofthe Group in Regulate and Attend condition separately (B and C). The scatterplots represent the changes in emotional experience in the Regulate and Attend conditions of individual subjects in the NF group and Sham group separately (D and E).

2.


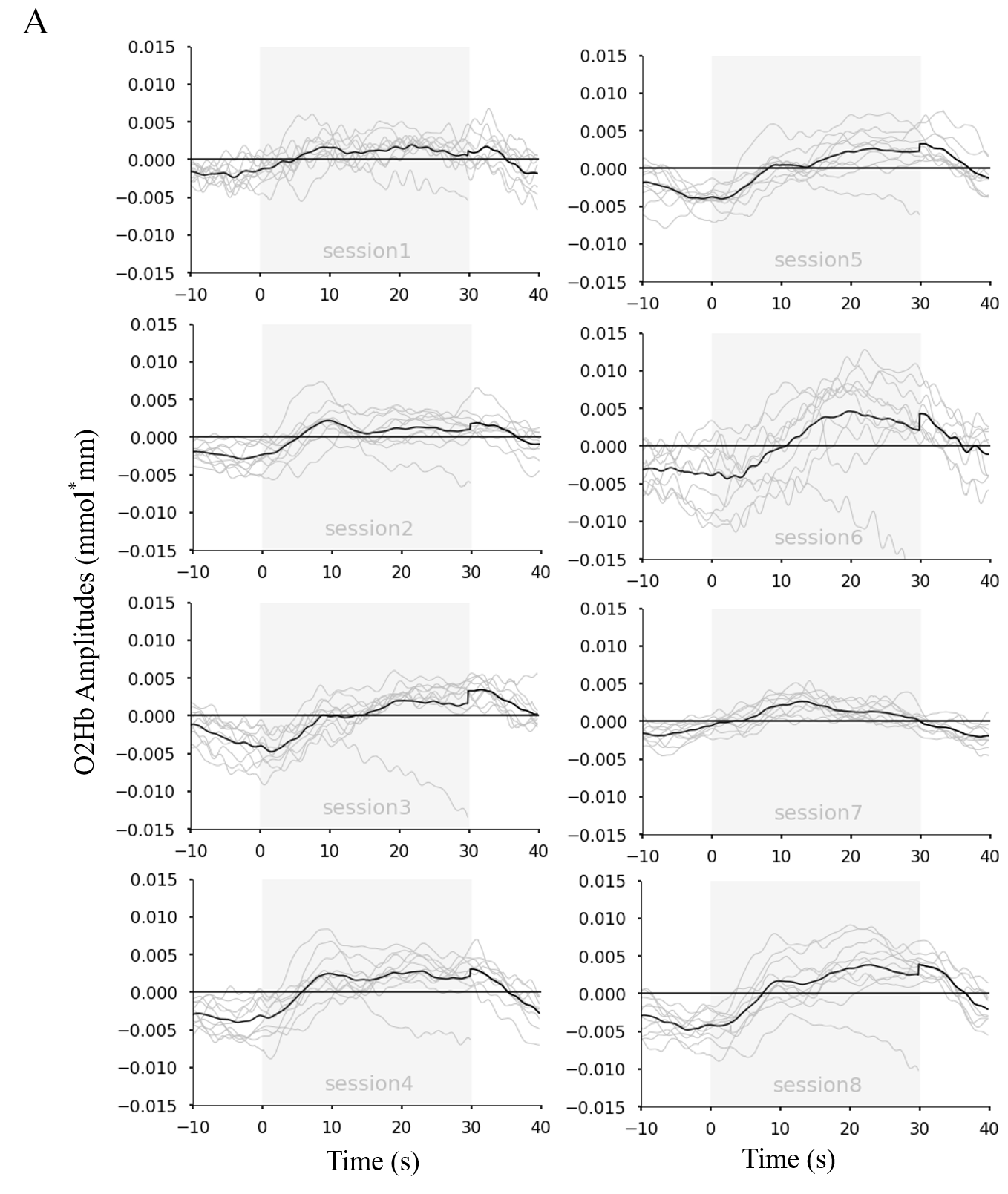


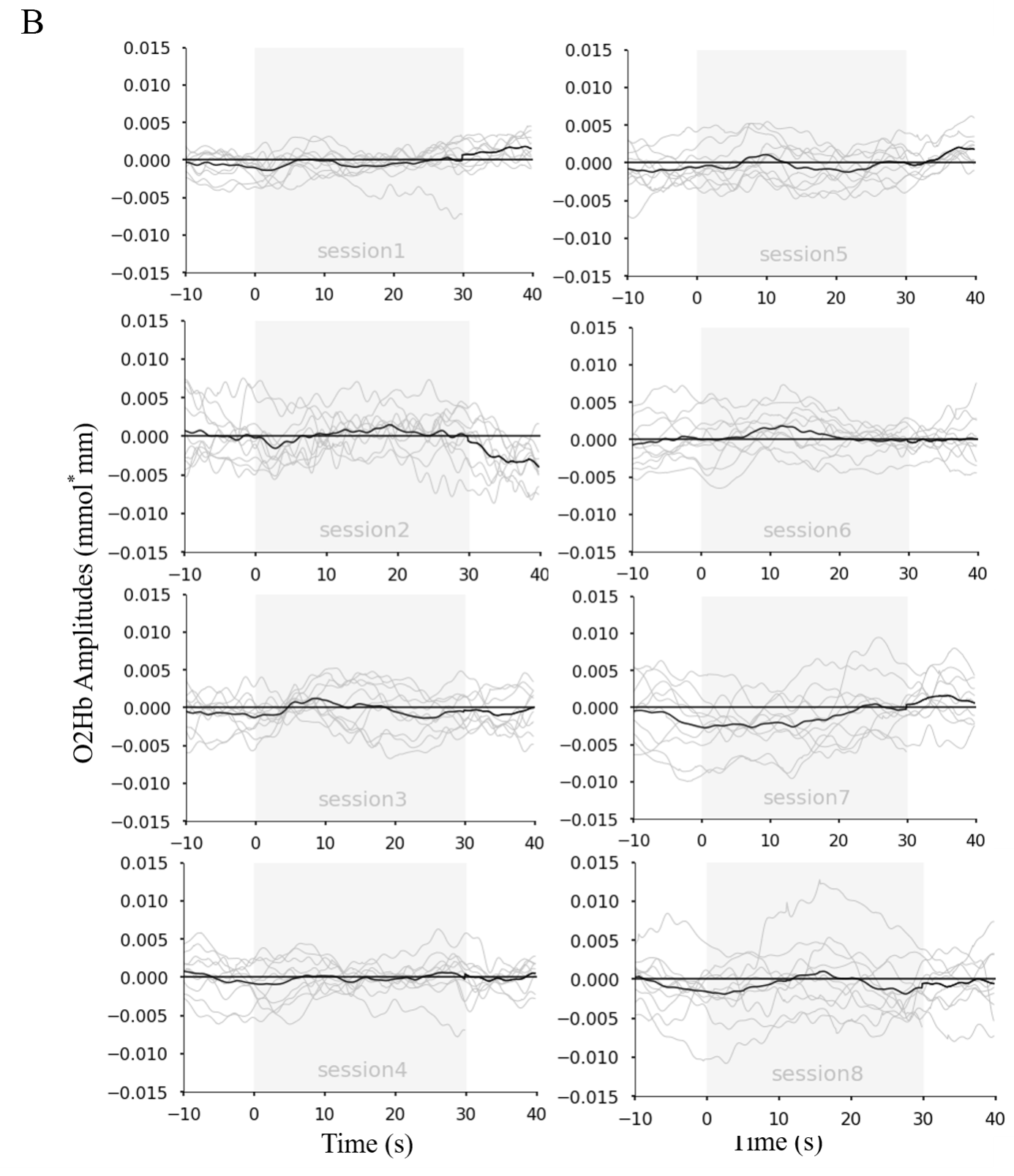


**Figure S2**.The graph shows the qualitative data analysis of pre-processed O2Hb amplitudes in target channel over the time course of eight NIRS-based neurofeedback sessions in all the participants of the NF group and Sham group separately (A and B). The part shaded in gray represents the upregulation phase of 30 s duration. The dashed line indicates the average amplitudes in each trial of the participants in the NF group (A) and Sham group (B), and the solid line indicates the mean amplitudes over one session.
